# Differential lncRNA/mRNA Expression Profiling and Functional Network Analyses in Bmp2 Deletion of Mouse Dental Papilla Cells

**DOI:** 10.3389/fgene.2021.702540

**Published:** 2021-12-22

**Authors:** Feng Wang, Ran Tao, Li Zhao, Xin-Hui Hao, Yi Zou, Qing Lin, Meng Meng Liu, Graham Goldman, Daoshu Luo, Shuo Chen

**Affiliations:** ^1^ Laboratory of Clinical Applied Anatomy, Department of Human Anatomy, School of Basic Medical Sciences, Fujian Medical University, Fuzhou, China; ^2^ Department of Developmental Dentistry, School of Dentistry, The University of Texas Health Science Center at San Antonio, San Antonio, TX, United States; ^3^ Greehey Children’s Cancer Research Institute, The University of Texas Health Science Center at San Antonio, San Antonio, TX, United States

**Keywords:** bone morphogenetic protein 2, dental mesenchymal papilla cells, coding and non-coding RNAs, bioinformatics, signal pathways

## Abstract

Bmp2 is essential for dentin development and formation. Bmp2 conditional knock-out (KO) mice display a similar tooth phenotype of dentinogenesis imperfecta (DGI). To elucidate a foundation for subsequent functional studies of cross talk between mRNAs and lncRNAs in Bmp2-mediated dentinogenesis, we investigated the profiling of lncRNAs and mRNAs using immortalized mouse dental Bmp2 flox/flox (iBmp2^fx/fx^) and Bmp2 knock-out (iBmp2^ko/ko^) papilla cells. RNA sequencing was implemented to study the expression of the lncRNAs and mRNAs. Quantitative real-time PCR (RT-qPCR) was used to validate expressions of lncRNAs and mRNAs. The Gene Ontology (GO) and Kyoto Encyclopedia of Genes and Genomes (KEGG) databases were used to predict functions of differentially expressed genes (DEGs). Protein–protein interaction (PPI) and lncRNA–mRNA co-expression network were analyzed by using bioinformatics methods. As a result, a total of 22 differentially expressed lncRNAs (16 downregulated vs 6 upregulated) and 227 differentially expressed mRNAs (133 downregulated vs. 94 upregulated) were identified in the iBmp2^ko/ko^ cells compared with those of the iBmp2^fx/fx^ cells. RT-qPCR results showed significantly differential expressions of several lncRNAs and mRNAs which were consistent with the RNA-seq data. GO and KEGG analyses showed differentially expressed genes were closely related to cell differentiation, transcriptional regulation, and developmentally relevant signaling pathways. Moreover, network-based bioinformatics analysis depicted the co-expression network between lncRNAs and mRNAs regulated by Bmp2 in mouse dental papilla cells and symmetrically analyzed the effect of Bmp2 during dentinogenesis via coding and non-coding RNA signaling.

## Introduction

Bone morphogenetic protein 2 (Bmp2) is a multiple-functional growth factor and is involved in many organ developments (Macias et al., 1997; Schlange et al., 2000; Ou et al., 2014). The bone morphogenetic proteins (BMPs) are structurally related to the transforming growth factor β (TGF-β) superfamily. The members of the BMP family play various biological functions during embryonic development (Hogan, 1996; Shi and Massagué, 2003; Wu et al., 2003; Chen et al., 2004; Miyazono et al., 2005), including a vital role in tooth development and formation (Meguro et al., 2019). Among the BMP family members, Bmp2 has been widely investigated for its diverse biological functions, particularly during dental cell differentiation (Casagrande et al., 2010; Wang et al., 2012; Yang et al., 2012; Guo et al., 2015; Yang et al., 2017; Malik et al., 2018). Bmp2 is expressed in mesenchymal cells and promotes mesenchymal progenitor/stem cell commitment to the odontoblast lineage by regulating a series of transcription factors and others (Yamashiro et al., 2003; Chen et al., 2005; Chen et al., 2008; Cho et al., 2010; Agas et al., 2013; Yang et al., 2017).

During tooth development, at the initiation stage (E10-12), the dental lamina is formed as an epithelial clustering, and the dental epithelium and mesenchyme are distinguished. The Bmp2 gene transcript is seen in those areas of the dental lamina where it started to form a bud (Aberg et al., 1997; Heikinheimo et al., 1998; Nadiri et al., 2004; Chen et al., 2008). At the bud stage (E12-13), Bmp2 expression is detectable in the dental epithelium and mesenchyme throughout the bud period. At the cap stage, Bmp2 expression is prominent at E14 and mainly localized at the epithelial enamel knot during the late cap stage, and Bmp2 expression expands to the neighboring inner dental epithelium where the secondary enamel knots will be formed and its signal is seen in dental mesenchyme. At the bell stage, Bmp2 expression is detected in the dental mesenchymal cells (Dong et al., 2014; Dong et al., 2016; Gao et al., 2018). Later, Bmp2 expression spreads to the dental papilla and is intense in the pre-odontoblasts. At the postnatal days (PN), Bmp2 is continually expressed in odontoblasts and ameloblasts and detected in the dental papilla as well as adjacent tissues including dental follicle, periodontal ligamental cells, cementoenamel junction, and Hertwig’s epithelial root sheath (HERS) and osteoblasts in alveolar bones (Yamashiro et al., 2003; Kémoun et al., 2007).

The Bmp2 binds to two distinct types, type I serine/threonine kinase and type II receptors, which are necessary for signal transduction (Shi and Massagué, 2003; Chen et al., 2004; Miyazono et al., 2005). The Bmp signal regulates downstream gene expression through either the canonical Smad or non-canonical Smad pathway. Following heterodimerization, type I receptors are phosphorylated by type II receptors and subsequently activate the receptor-regulated R-Smad-1/-5/-8 through phosphorylation (Bhatt et al., 2013; Graf et al., 2016). The phosphorylated Smad-1/-5/-8 heterodimers form a complex with the common mediator Smad-4 (Co-Smad-4). Following nuclear translocation of the R-Smad-1/-5/-8/Co-Smad-4 complex, Bmp target gene expression is induced (Bhatt et al., 2013). On the other hand, Bmp2 also activates non-canonical Smad signaling pathways such as mitogen-activated protein kinases (MAPKs), c-Jun amino-terminal kinase (JNK), phosphoinositol-3 kinase (PI3K), Akt, and small GTPases (Derynck and Zhang, 2003). Thus, these canonical Smad pathways cooperate with non-canonical Smad pathways to regulate various cellular responses. Also, Bmp2 can crosstalk with other factors, such as Wnt, Fgf, and other factors, regulating cell proliferation, differentiation, and tissue development (Guo and Wang, 2009; O'Connell et al., 2012; Agas et al., 2013; Saito et al., 2016; Wu et al., 2016; Zhang et al., 2016; Chakka et al., 2020).

Global Bmp2 knockout (KO) mice are nonviable. Homozygous Bmp2 mutant embryos die at embryonic day 9.5 (E9.5) and exhibited defects in cardiac development, manifested by the abnormal development of the heart in the exocoelomic cavity (Zhang and Bradley, 1996). However, Bmp2 conditional KO mice were generated and viable. Teeth with Bmp2 cKO mice display the similar phenotype of dentin defects to that of dentinogenesis imperfecta (DGI) in humans and mice, the most common dentin genetic diseases, showing retardation of tooth growth, abnormal dentin structure with wide predentin, and thin dentin (Zhang et al., 2001; Lu et al., 2007; Lee et al., 2009). Accumulating evidence indicated that Bmp2 is capable of regulating a lot of bone/dentin-related gene expressions and transcriptional factors as well as dental cell proliferation and differentiation in tooth development (Chen et al., 2008; Cho et al., 2010; Oh et al., 2012; Yang et al., 2017). Additionally, cross talk has been described between Bmp signaling–associated mRNAs and non-coding RNAs during dental cell proliferation and differentiation (Wang F. et al., 2017; Wang J. et al., 2017; Liu et al., 2018; Liao et al., 2020; He et al., 2021).

High-throughput sequencing techniques have revolutionized our understanding of the human genome (Kapranov et al., 2007). The whole transcriptome study has demonstrated that although more than 90% of the genome can be transcribed into RNAs, only approximately 2% of the human genome contains protein-coding regions (Kapranov et al., 2007; Jalali et al., 2016). The remaining non-coding regions are transcribed into large amounts of non-coding RNAs (Jalali et al., 2016). lncRNAs are the largest class of non-coding RNAs composed of more than 200 nucleotides to over 100 kb in length, which lack protein-coding potential (Ponting et al., 2009). The lncRNAs comprise thousands of transcripts with distinct biogenesis, subcellular localization, and molecular functions (Chen, 2016). Studies have suggested that lncRNA genome complexity plays biological roles in regulating gene expression at transcriptional, post-transcriptional, and epigenetic levels in a lot of cellular and biological processes, such as signal transduction, cell proliferation, differentiation, and organ development (Batista and Chang, 2013; Fatica and Bozzoni, 2014; Quinn and Chang, 2016; Bunch, 2018; Statello et al., 2021). Therefore, alteration of lncRNA expression is related to numerous diseases (Fatica and Bozzoni, 2014; Wu and Du, 2017). lncRNAs can regulate the expression of growth factors, transcriptional and epigenetic factors, and vice versa (Yang et al., 2014). The pattern of cell- and tissue-specific lncRNA expression provides new insights into tissue development, diagnosis, and treatment of several diseases (Chen et al., 2021). Up to date, over 100,000 lncRNAs have been identified in the human genome and new lncRNAs are being discovered and characterized rapidly (Zhao et al., 2021).

However, the interplay of Bmp2-mediated mRNAs and lncRNAs in the regulation of odontoblastic differentiation and function has not completely been understood. Previously, our group generated immortalized mouse dental Bmp2 flox/flox (iBmp2^fx/fx^) and Bmp2 knock-out (iBmp2^ko/ko^) papilla cells. In this study, we detected the differential lncRNA and mRNA expression profiles in these two types of dental papilla cell lines by RNA sequencing. A network-based bioinformatics analysis was performed to investigate the cross talk between lncRNAs and mRNAs via integrating lncRNA–mRNA interactions, gene co-expression, and protein–protein interactions. Our results demonstrated that the synergistic or competitive lncRNA–mRNA cross talk may play an important role in Bmp2-mediated odontoblastic differentiation.

## Materials and Methods

### *In Situ* Alkaline Phosphatase Assay and Alizarin Red S Staining

Mouse dental iBmp2^fx/fx^ and iBmp2^ko/ko^ papilla cells were generated by our group as previously described (Wu et al., 2010; Wu et al., 2015). To induce cell differentiation, the cells were cultured in α-MEM supplemented with 10% fetal bovine serum (FBS), 1% antibiotics, 10 mM sodium β-glycerophosphate, 50 μg/ml ascorbic acid, and 100 nM dexamethasone for 7 and 10 days. Then, the cells were fixed and washed in PBS. *In situ* alkaline phosphatase (ALP) assay was carried out in accordance with the instructions. For cell mineralization assessment, the cells were fixed in 10% formalin as well as were treated with 1% Alizarin Red S dye (pH 4.2).

### RNA-Seq and Gene Expression Analysis

The iBmp2^fx/fx^ and iBmp2^ko/ko^ cells were cultured in 100-mm^2^ dishes to 80% confluence. The cells were harvested after 6, 48, or 72 h, and RNAs were extracted using TRIzol reagent (QIAGEN Inc.). The RNA quality was assessed using a bio-analyzer. The RNA-seq libraries were prepared from total RNAs in accordance with Illumina’s RNA specimen preparation protocol (Illumina Inc. San Diego, CA, United States). Paired reads to the UCSC mm9 genome build were mapped by a TopHat2 aligner. To quantify gene expression, HTSeq was used to obtain raw read counts per gene and then converted to RPKM in accordance with the gene length and total mapped read count per sample. The DEGs were calculated, as gene expression levels were measured by Log_2_-transformed RPKM (|log_2_ fold Change| >1, *p* value <0.05). RNA-seq data were submitted and deposited to The Gene Expression Omnibus (GEO, http://www.ncbi.nlm.nih.gov/geo/). The GEO accession number is GSE174429.

### Gene Ontology (GO) and Kyoto Encyclopedia of Genes and Genomes (KEGG) Analysis

GO analysis was used to investigate the roles of all differentially expressed mRNAs (https://david.ncifcrf.gov/). DAVID-based KEGG analysis was used to determine the significant pathways related to the differentially expressed mRNAs. Fisher’s exact test and the x^2^ test were used to select the significant GO categories and pathways. The threshold of significance was a *p* value < 0.05, and a false discovery rate (FDR) was calculated to correct the *p* value.

### Generation of the Protein–Protein Interaction (PPI) Network

The differentially expressed genes (DEGs) were imported into a STRING database, and the species was limited to “Mus musculus” to obtain PPI information. The network relationships with high confidence (≥0.75) were screened and imported into Cytoscape 3.6.2 to draw a PPI network diagram. According to the calculation method of the MCC (maximum clique centrality) algorithm, the top 20 hub genes were screened by using the “cytoHubba” plug-in. Additionally, the interactive density region was extracted using the “MCODE” plug-in in Cytoscape.

### Construction of the lncRNA–mRNA Co-Expression Network

The mRNA–lncRNA co-expression network, which was used to identify interactions between the differentially expressed lncRNAs and mRNAs, was constructed based on Pearson’s correlation analysis. For each pair of genes, correlation coefficients of 0.95 or greater were selected to construct the network using the OmicStudio tools at https://www.omicstudio.cn/tool. In the network, each mRNA or lncRNA corresponds to a node, and the nodes are connected by edges.

### Quantitative Reverse Transcription PCR (RT-qPCR)

Total RNA samples were isolated with TRIzol (Invitrogen, California, United States) and reverse transcribed into cDNA with the Prime Script RT reagent kit with gDNA Eraser (RR047A) following the manufacturers’ guidelines (Takara, Japan). The RNA was reverse transcribed into cDNA at 37°C for 15 min, and the reaction was stopped at 85°C for 5 s. RT-qPCR was performed using the SYBR™ Select Master Mix (Applied Biosystems, California, United States) on a CFX96 system (Bio-Rad, California, United States). The relative gene expression was calculated with the 2^−ΔΔCt^ method using glyceraldehyde-3-phosphate dehydrogenase (GAPDH) as the reference housekeeping gene. The RT-qPCR primers are listed in Table 1.

**TABLE1:** RT-qPCR primers.

**Gene**	**Forward primer (5’ --3′)**	**Reverse primer (5’-- 3′)**
Fgf2	GCG​ACC​CAC​ACG​TCA​AAC​TA	TCC​CTT​GAT​AGA​CAC​AAC​TCC​TC
Id2	ATG​AAA​GCC​TTC​AGT​CCG​GTG	AGC​AGA​CTC​ATC​GGG​TCG​T
Id3	CTG​TCG​GAA​CGT​AGC​CTG​G	GTG​GTT​CAT​GTC​GTC​CAA​GAG
Lhx1	CCA​AGC​GAT​CTG​GTT​CGC​A	CCG​GAG​ATA​AAC​TAG​GGT​CAC​TG
Smad7	GAC​AGC​TCA​ATT​CGG​ACA​ACA	CAG​TGT​GGC​GGA​CTT​GAT​GA
Tcf7	ACA​GTG​CTC​TAG​GCT​GTC​C	CGA​CCT​GAG​AAT​GTT​GGT​GCT
Wnt1	TTC​GGC​AAG​ATC​GTC​AAC​CG	GCC​AAA​GAG​GCG​ACC​AAA​ATC
Wnt11	GCA​CTG​AAT​CAG​ACG​CAA​CAC	CGA​CAG​GGC​ATA​CAC​GAA​GG
Klf10	AGT​GTC​ATC​CGT​CAC​ACA​GC	CAC​TGC​AGC​ACA​GGG​TAT​GT
Lnc87211.1	GCC​TCC​CAG​AGA​AGT​GTG​AA	ATA​CAC​ACT​GCA​GAC​AGC​TAC​A
Lnc86888.1	GTG​CGC​ATA​TCA​CAG​TGT​CG	AAC​AGG​AAA​TCA​CTC​GCC​GT
Lnc87189.1	CTT​GGT​GTC​CTT​GGA​TGA​CCT	GCA​TAA​GGA​AAG​AGA​GCC​ACC
Gapdh	AGG​TCG​GTG​TGA​ACG​GAT​TTG	TGT​AGA​CCA​TGT​AGT​TGA​GGT​CA

### Statistical Analysis

Data were presented as the mean ± SEM. RT-qPCR validation data presented were analyzed with Student’s *t* test. *p < 0.05* was considered statistically significant. Significant differences are noted by asterisks, with single asterisks representing *p < 0.05*, two asterisks representing *p < 0.01*, three asterisks representing *p < 0.001*, and four asterisks representing *p < 0.0001.*

## Results

### Deletion of Bmp2 Causes Delay of Dental Papilla Mesenchymal Cell Differentiation and Mineralization

**FIGURE 1:**
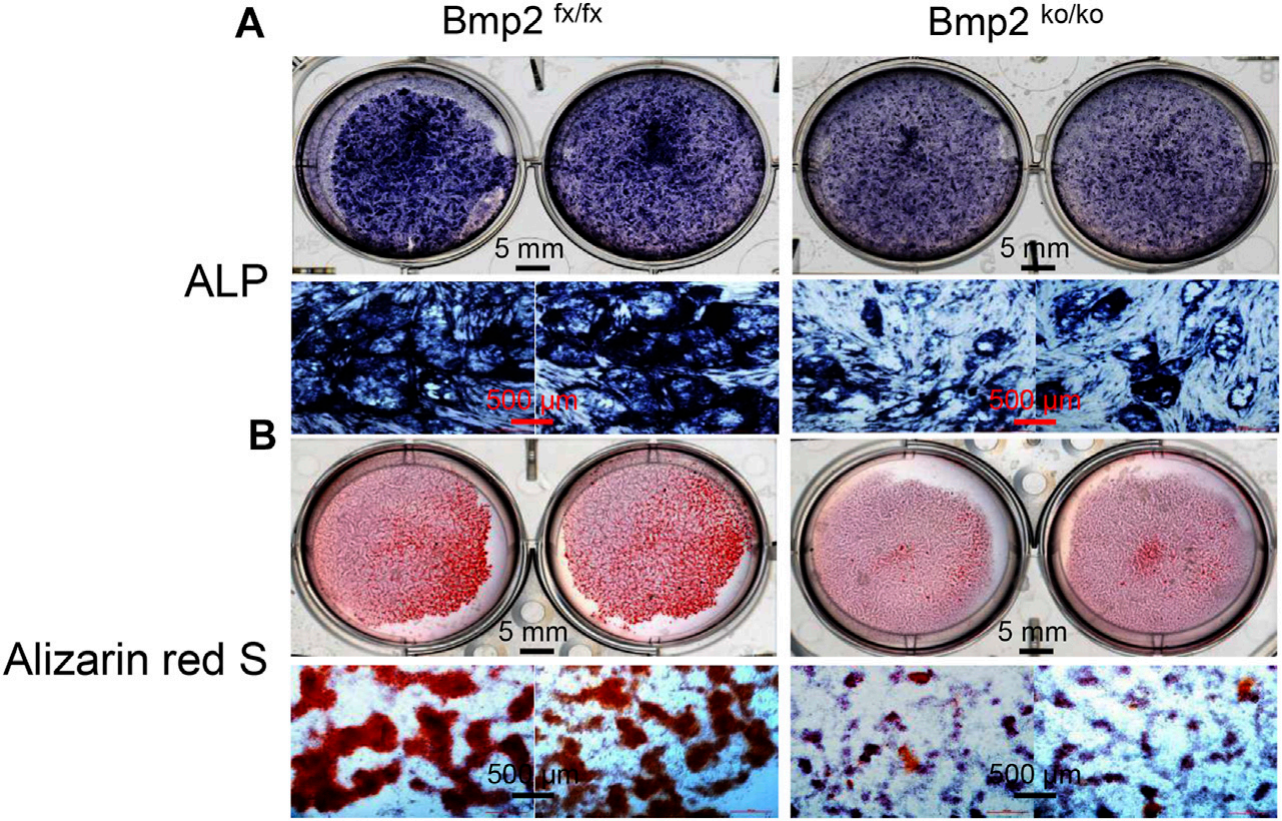
Deletion of Bmp2 delays mouse dental papilla cell differentiation and mineralization. **(A)** iBmp2^fx/fx^ and iBmp2^ko/ko^ cells were cultured in the calcifying medium for 7 days. ALP activity was analyzed using *in situ* ALP staining. **(B)** For cell mineralization assay, both the iBmp2^fx/fx^ and iBmp2^ko/ko^ cells were treated with the calcifying medium for 10 days. Mineralized nodules were visualized with Alizarin Red S staining. fx, floxed; ko, knock-out.

To determine the effect of Bmp2 on dental papilla cell differentiation and mineralization activities, we measured the alkaline phosphatase (ALP) activity by *in situ* ALP histochemistry since ALP is a marker of dental cell differentiation. Both cells were cultured in the calcifying medium in given time periods. This result showed delayed dental papilla cell differentiation in the iBmp2^ko/ko^ cells compared to that in the Bmp2^fx/fx^ cells. Additionally, deletion of the *Bmp2* gene led to low activity of the dental papilla cell mineralization as observed through Alizarin Red S staining (Figure 1).

### Overview of Differential lncRNA/mRNA Expression Profiling

**FIGURE 2:**
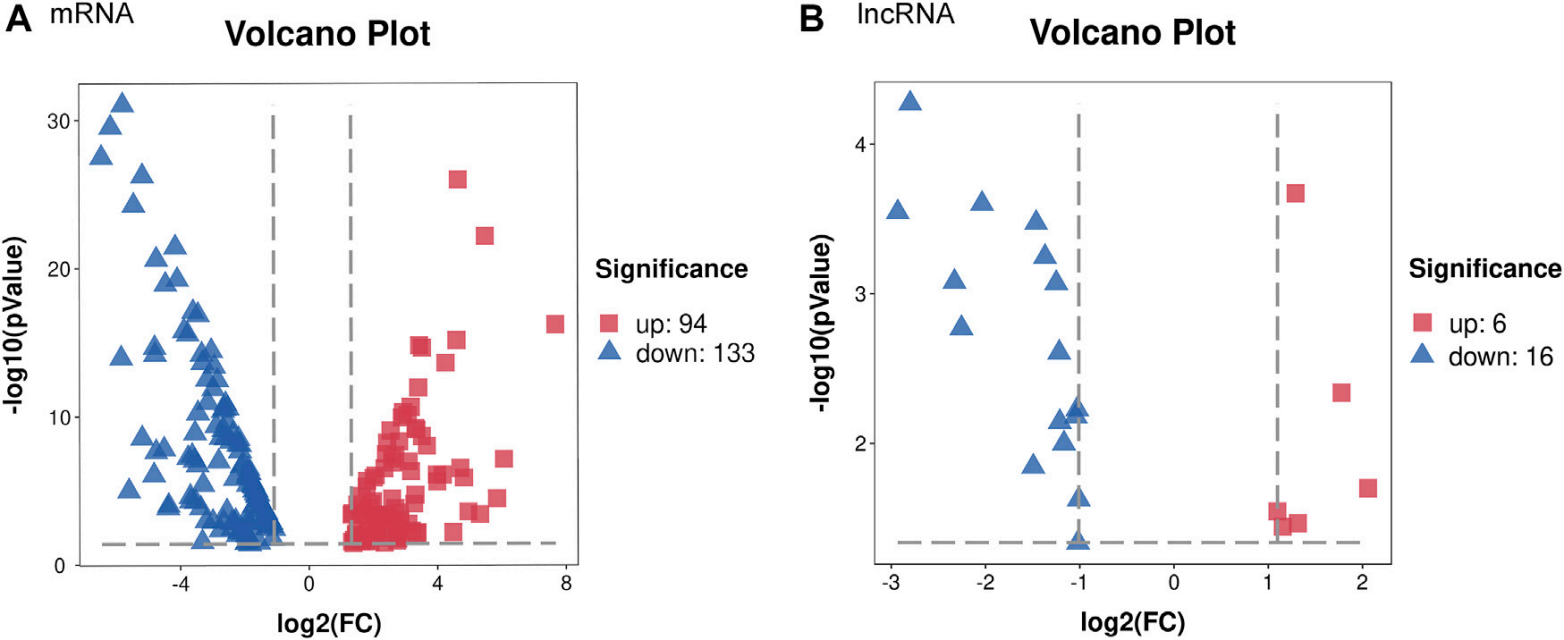
Differentially expressed genes between the iBmp2^fx/fx^ and iBmp2^ko/ko^ groups. Our data showed that 94 mRNAs upregulated and 133 mRNAs downregulated in the iBmp2^ko/ko^ cells compared to those in the iBmp2^fx/fx^ cells **(A)**. Meanwhile, 6 lncRNAs upregulated and 16 lncRNAs downregulated (|log2FC| ≥1; Qvalue <=0.05) in the iBmp2^ko/ko^ cells **(B)**. Red represents upregulated DEG, blue represents downregulated DEG.

**FIGURE 3:**
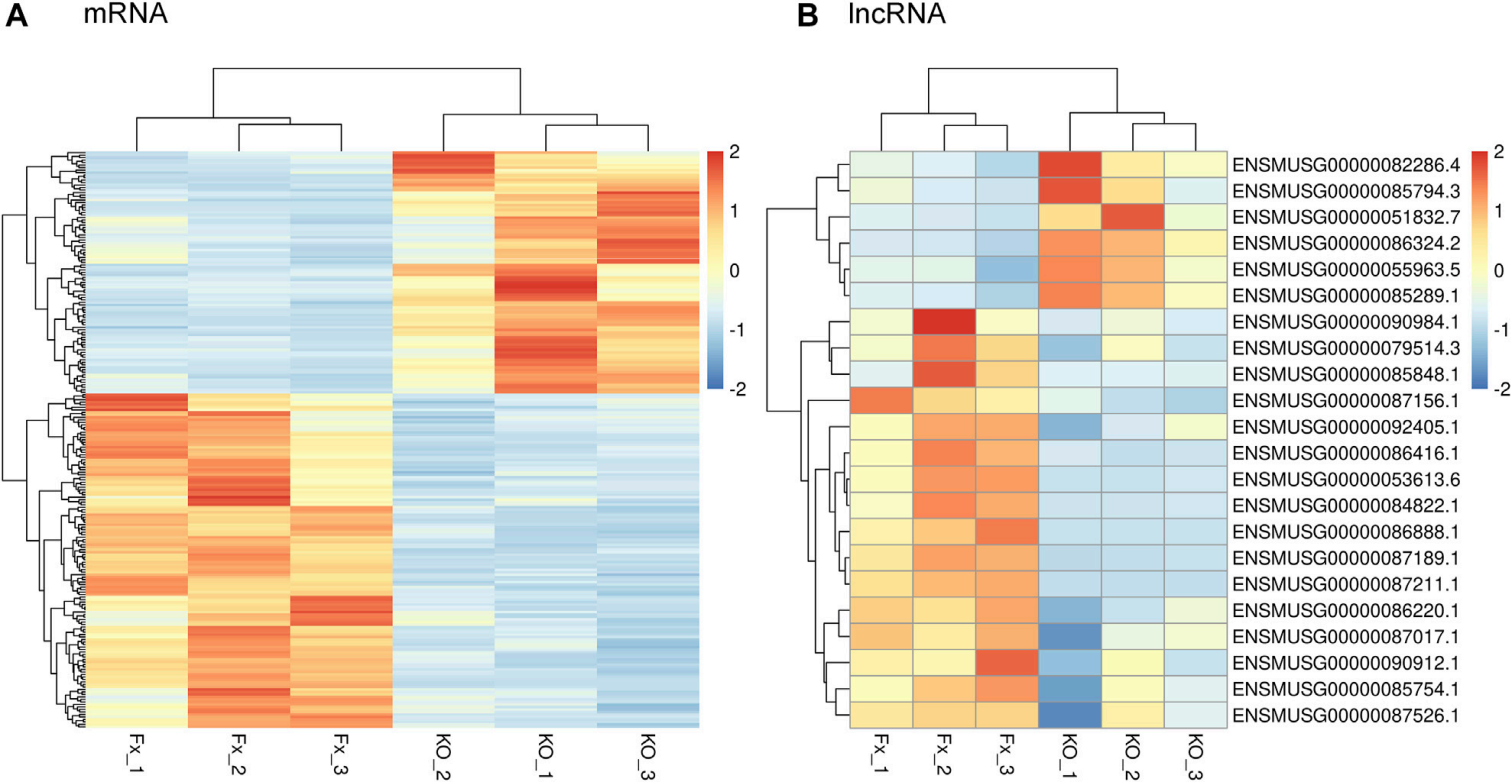
Genetic clustering of differentially expression genes (|log2FC| ≥1; Q value <=0.001). **(A)** mRNAs; **(B)** and lncRNAs. The red color represents high fold-change, and the blue color represents low fold-change.

To identify DEGs between the iBmp2^fx/fx^ and iBmp2^ko/ko^ cells, we performed and analyzed the RNA-seq data by using the limma package. The cutoff criteria were as follows: |log fold change| (the absolute value of log2 in the fold change of gene expression) > 1 and *p* value <0.05. Our data demonstrated that 94 mRNAs were upregulated, while 133 mRNAs were downregulated in the iBmp2^ko/ko^ cells compared with those in the iBmp2^fx/fx^ cells (Figure 2A). Likewise, 6 lncRNAs were upregulated whereas 16 lncRNAs were downregulated in the iBmp2^ko/ko^ cells compared to those in the iBmp2^fx/fx^ cells (Figure 2B). The volcano maps of mRNA and lncRNA DEGs were drawn by the ggplot2 package. The heatmaps of mRNA and lncRNA DEGs were shown by the heatmap package (Figure 3).

### Pathway and GO Analyses of Differentially Expressed mRNAs

**FIGURE 4:**
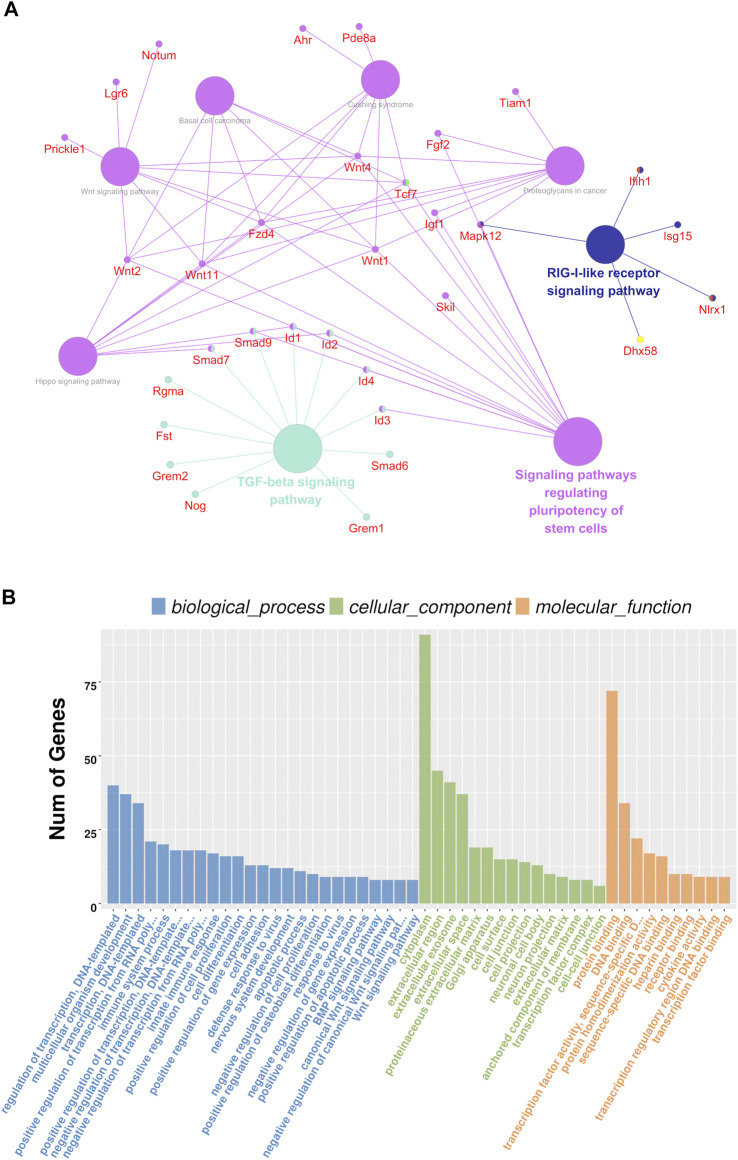
Analysis results of KEGG and GO analyses. **(A)** Possible signaling pathways are represented by circles, and the involved genes are represented by red characters. The possible signaling pathways showed the larger diameters of the circle; the larger the diameters are the more significant the signal pathway is. **(B)** GO analysis in a cellular component, biological process, and molecular function.

The KEGG analysis showed that the DEGs were largely enriched in the TGF-β/BMP signaling pathway, Hippo signaling pathway, Wnt signaling pathway, and signaling pathways regulating pluripotency of stem cells (Figure 4A). These signal pathways are cross talks and are involved in dental and other cell proliferation and differentiation via regulating transcriptional and growth factors including *Id2*, *Id3*, *Fgf2*, *Klf10*, *Lhx1*, *Smad7*, *Tcf7*, *Wnt1*, and *Wnt11* (Ho et al., 2011; Koizumi et al., 2013; Sternberg et al., 2013; Bakopoulou et al., 2015; Chen et al., 2016; Zhang et al., 2016; Wang and Martin, 2017; Neves and Sharpe, 2018; Chen et al., 2019; Liu et al., 2019; Chakka et al., 2020; Niki et al., 2021).

The GO analysis showed that the following biological pathways (BPs) were notably enriched among the DEGs: regulation of gene transcription, regulation of transcription from RNA polymerase II promoter, multicellular organism development, positive regulation of cell proliferation, cell differentiation, Wnt signaling, and BMP signaling pathways, etc. (Figure 4B). Besides, the following cellular components (CCs) were found to be largely enriched in the extracellular exosome, proteinaceous extracellular matrix, anchored component of the membrane and transcription factor complex, cell–cell junction, and cell protection (Figure 4B). Additionally, the following molecular functions (MFs) were largely enriched in protein binding, transcription factor activity, sequence-specific DNA binding, receptor binding, and transcription regulatory region DNA binding (Figure 4B). This result suggests that Bmp2 and other signaling pathways regulate dental cell proliferation and cell differentiation as well as tissue development through those aforementioned pathways (Graf et al., 2016; Salazar et al., 2016; Wu et al., 2016; Migliorini et al., 2020).

### Analysis Results of the Protein–Protein Interaction (PPI) Network

**FIGURE 5:**
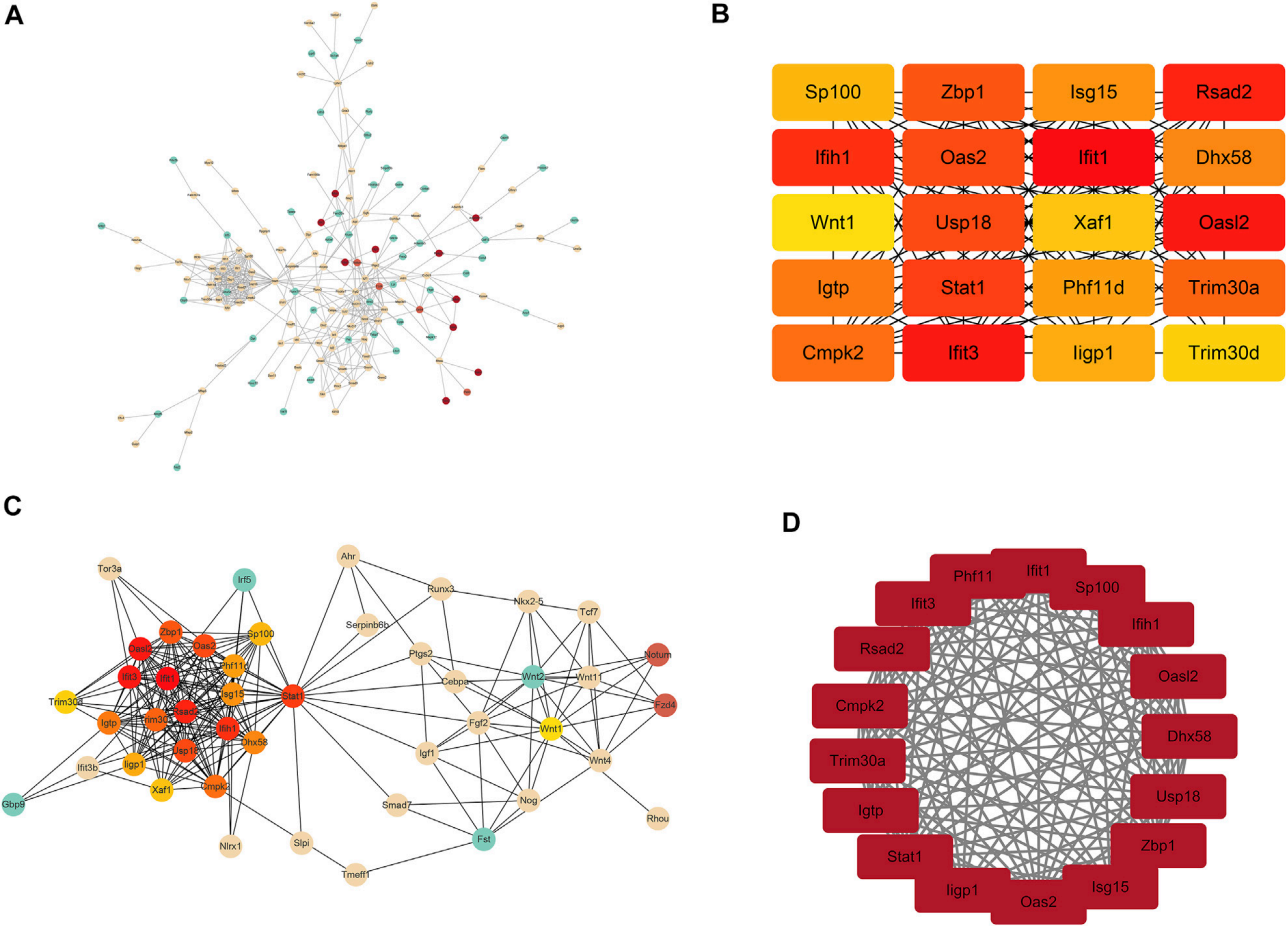
Analysis results of PPI. **(A)** Total network interaction of all DEGs. The redder the color, the more important it is in the network relationship. **(B)** Top 20 hub genes in the interaction network. **(C)** Total network interaction of the top 20 hub genes and the differential genes that are directly related to them. **(D)** Top dense regions of the interactive density region in the PPI network.

To gain more insights into the role of these 227 genes in the Bmp2 network and find the hub genes, which were significantly implicated in, a PPI network based on these 227 genes (94 upregulated genes and 133 downregulated genes) was established on the STRING database for functional association analysis, and the sequential visualization was performed on Cytoscape (Figure 5A). The plug-in “cytoHubba” determined the top 20 hub genes by MCC algorithm (Figure 5B) and related the differential genes according to the association grade difference between different genes (Figure 5C). The interactive density region in the PPI network by “MCODE’’ plug-in was also discovered. The figure below showed the top dense regions (Figure 5D). The PPI analysis predicted that the 9 Bmp2-induced proteins tested can interact with each other. For instance, Fgf2 binds to Id3 and to Wnt11, Lhx1 to Wnt11, Smad7 to Klf10 and to Id2, Tcf7 to Wnt 1 and Id2, etc. On the other hand, one protein interacts with another *via* a third protein. For example, Id2 binds to Klf10 *via* Smad7; Lhx1 binds to Wnt1 *via* Wnt11 and Tcf7 *via* Wnt11; and Fgf2 binds to Id2 *via* Tcf7 and Lhx1 *via* Wnt11 as well as others. This study suggests that these proteins play synergistic roles in the regulation of dental cell proliferation and differentiation.

### Analysis Results of Co-Expression Networks Between mRNAs and lncRNAs

**FIGURE 6:**
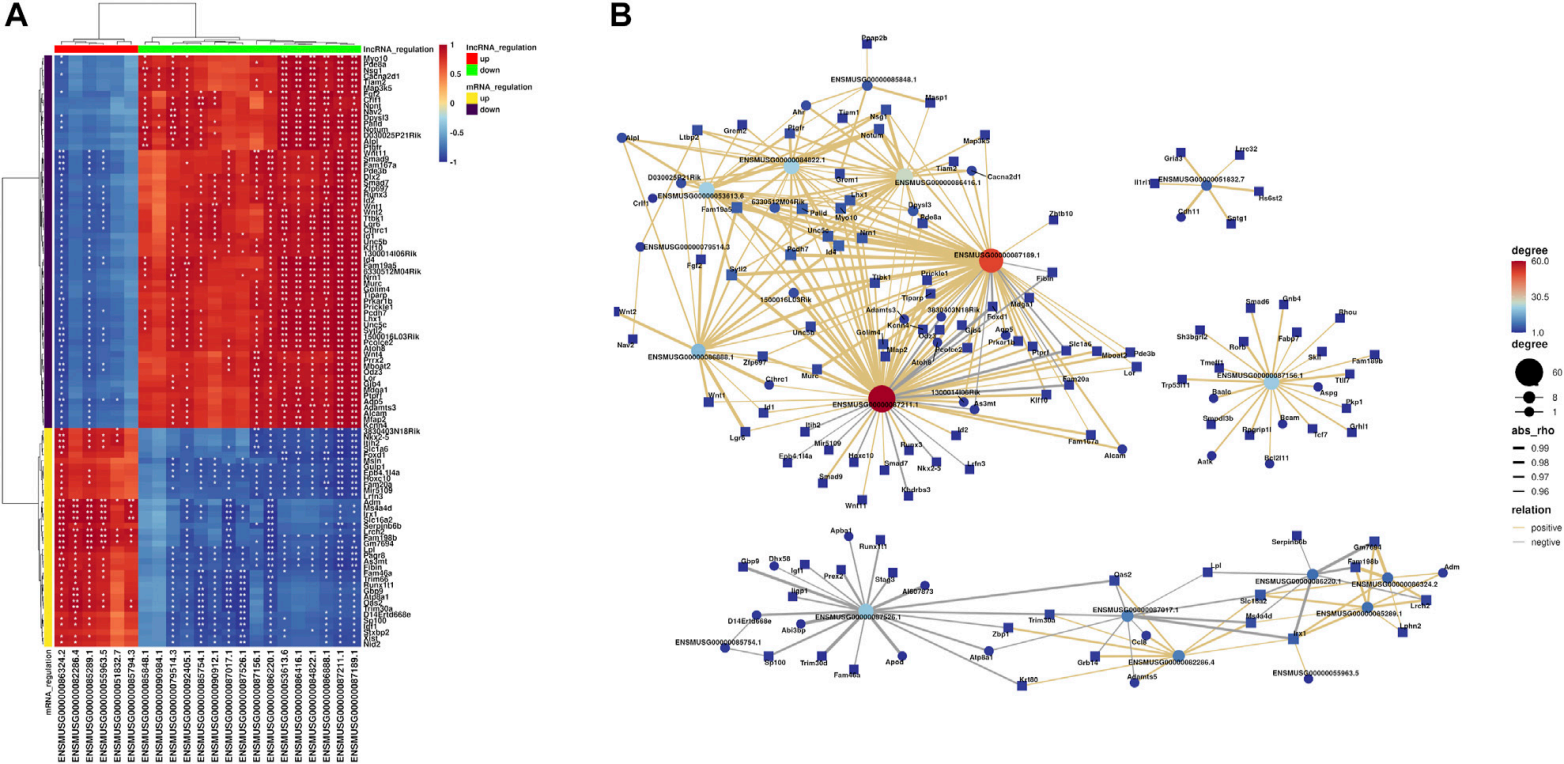
Results of co-expression networks between mRNAs and lncRNAs. **(A)** Heatmaps are used to describe the relationship between lncRNAs and mRNAs (different colors of lncRNA or mRNA regulation). The differences that are statistically significant are denoted by asterisks, with single asterisks representing *p* < 0.05 and two asterisks representing *p* < 0.01. **(B)** Hub co-expression network between lncRNAs and mRNAs. The yellow line represents the positive regulation, the gray line represents the negative regulation, and the thickness of the line represents the relative strength. In addition, the size and color depth of the points represent the number of related genes.

Based on correlations between the differential expression levels of lncRNAs and mRNAs, a coding–noncoding gene co-expression network was established. We calculated the Pearson correlation coefficient (PCC) and used the R-value to calculate the correlation coefficient of the PCC between lncRNAs and mRNAs (lncRNA–mRNA PCC, not including lncRNA–lncRNA or mRNA–mRNA PCC). Those lncRNAs and mRNAs that had Pearson correlation coefficients (PCCs) ≥ 0.95 were selected to construct the co-expression network. The figure below showed the top 100 pairs of co-expression relationships (Figure 6A). Furthermore, visualization of the co-expression network between lncRNA and mRNAs was performed using the OmicStudio tools at https://www.omicstudio.cn/tool (Figure 6B). For instance, lncRNA ENSMUSG00000087211.1 (87,211.1) co-expressed with *Id2*, *Klf10*, *Smad7*, *Wnt1*, *Wnt11*, and *Lhx1*, while lncRNA ENSMUSG00000087189.1 (87,189.1) co-expressed with *Lhx1* and *Klf10*, lncRNA ENSMUSG00000086888.1 (86,888.1) co-expressed with *Fgf2* and *Wnt1*, and lncRNA ENSMUSG00000053613.6 (53,613.6) co-expressed with *Fgf2* and *Lhx1*. Although the co-expression of lncRNAs and mRNAs was identified in the dental papilla cells, the effect of cross talk between the mRNAs and lncRNA during dentinogenesis is required for further study.

### Validation of Differentially Expressed lncRNAs and mRNAs

**FIGURE 7:**
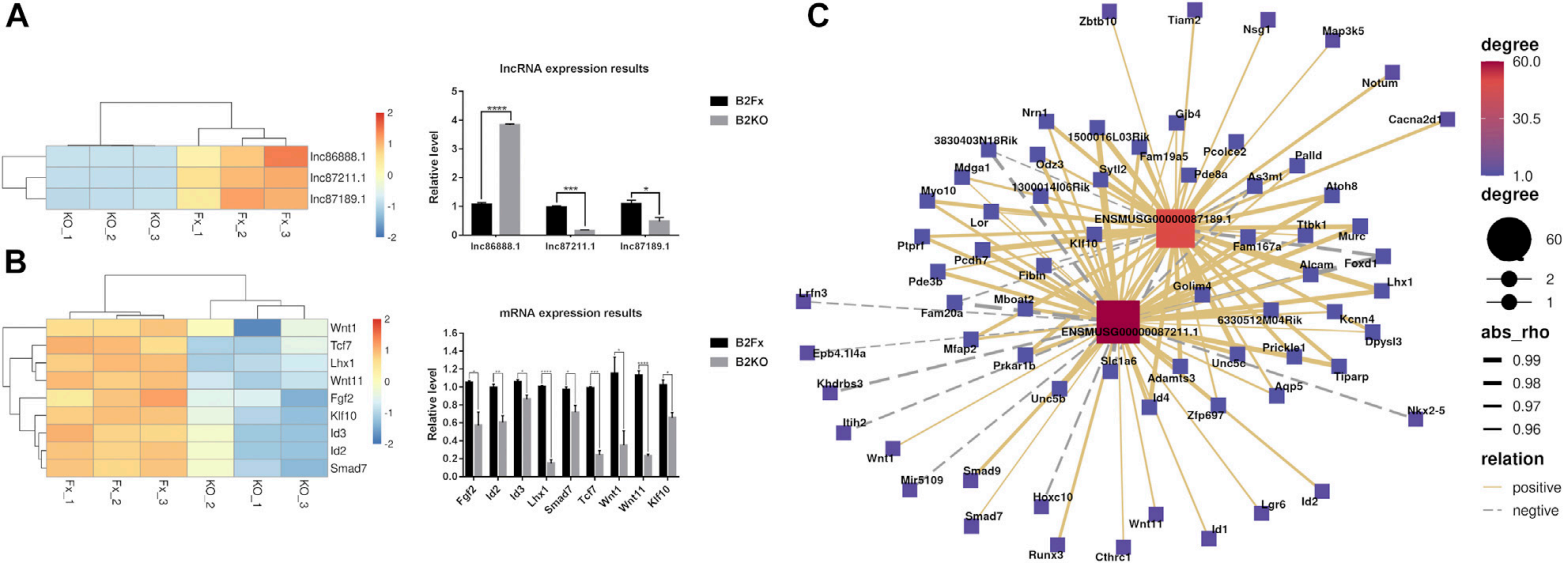
RT-qPCR relative expression results. There are 9 mRNAs and 3 hub lncRNAs validated by RT-qPCR. **(A)** Results of the RNA-seq and RT-qPCR from the lncRNAs of lncRNA87211.1 (ENSMUSG00000087211.1), lncRNA87189.1 (ENSMUSG00000087189.1), and lncRNA86888.1 (ENSMUSG00000086888.1). **(B)** Results of the RNA-seq and RT-qPCR from 9 mRNAs including *Fgf2*, *Id2*, *Id3*, *Klf10*, *Lhx1*, *Smad7*, *Tcf7*, *Wnt1*, and *Wnt11*. The red color represents high fold-change, and the blue color represents low fold-change. The abscissa is the gene name, and the ordinate is the relative expression of the genes. **(C)** Hub co-expression network between two lncRNAs lncRNA87211.1 and lncRNA87189.1 and 6 mRNAs. The solid line shows positive regulation while the dashed line represents negative regulation.

In order to verify the transcriptome sequencing results between the iBmp2^fx/fx^ and iBmp2^ko/ko^ cells and further analyze the possible signaling network mediated by Bmp2, the 9 representative mRNAs and 3 lncRNAs were selected for validation by using RT-qPCR since the 9 Bmp signal-associated mRNAs are involved in the dental cell proliferation, differentiation, and tooth development (Ho et al., 2011; Koizumi et al., 2013; Sternberg et al., 2013; Bakopoulou et al., 2015; Chen et al., 2016; Zhang et al., 2016; Chen et al., 2019; Liu et al., 2019; Chakka et al., 2020), and three novel lncRNAs (87,211.1, 87,189.1, and 86,888.1) co-expressed with these mRNAs (Figure 6B). The RT-qPCR results showed that the expression of lncRNA87211.1 and lncRNA87189.1 decreased in the iBmp2^ko/ko^ cells, as same as the results of RNA-seq, but lncRNA86888.1 expression was increased in the iBmp2^ko/ko^ cells which was opposite to the results of RNA-seq (Figure 7A). The mRNA expression of *Fgf2*, *Id2*, *Id3*, *Lhx1*, *Smad7*, *Tcf7*, *Wnt1*, and *Wnt11* by RT-qPCR analysis was consistent with the result of the RNA-seq (Figure 7B). Furthermore, the 6 validated mRNAs (*Id2*, *Klf10*, *Lhx1*, *Smad7*, *Wnt1*, *and Wnt11*) were positively correlated with the expression of lncRNA 87211.1 and lncRNA 87189.1, which was in accordance with the predicted lncRNA–mRNA co-expression network (Figure 7C). Accordingly, the PCC can be used to predict the relation network between mRNAs and lncRNAs.

## Discussion

Bone morphogenetic proteins (BMPs), members of the TGF-β superfamily, are multiple regulators for organogenesis and homeostasis. Among them, Bmp2 has been proven to be able to induce osteogenesis or dentinogenesis, independently (Bais et al., 2009; Yang et al., 2012). Several canonical and non-canonical Bmp2 signaling pathways have been reported involved in odontogenesis (Cho et al., 2010; Qin et al., 2012a; Qin et al., 2012b; Washio et al., 2012; Qin et al., 2014). Recently accumulated evidence has demonstrated that lncRNAs play biological roles in dental cell proliferation, cell proliferation, and tooth development through the regulation of growth and transcriptional factors (Zheng and Jia, 2016; Liu et al., 2018; Gil and Ulitsky, 2020; Wang et al., 2020; Li et al., 2021; Mirzadeh Azad et al., 2021). The cross talk between the Bmp2 signaling pathway and lncRNAs in dental cell proliferation, cell differentiation, and odontogenesis remains largely unclear. In the present study, we, for the first time, sequenced transcriptome in the iBmp2^fx/fx^ and iBmp2^ko/ko^ cells and performed bioinformatics analysis to study Bmp2-mediated signaling pathways and downstream molecules as well as to predict the co-expression network between lncRNAs and mRNAs regulated by Bmp2 *in vitro*.

Our data indicated that 94 mRNAs were upregulated, and 133 mRNAs were downregulated. Moreover, 6 lncRNAs were upregulated and 16 lncRNAs were downregulated by RNA-seq from the iBmp2 ^ko/ko^ and iBmp2 ^fx/fx^ papilla cells. GO analysis indicated that the DEGs were closely related to cell proliferation, cell differentiation, transcriptional regulation, protein–protein interactions, BMP- and Wnt-signal pathways, and so forth. Through KEGG analysis, these DEGs were found to be largely enriched in developmentally signaling pathways including TGF-β/BMP signaling pathway, Hippo signaling pathway, Wnt signaling pathway, and signaling pathways regulating pluripotency of stem cells, suggesting that Bmp2 might mediate cross-talk between these signaling pathways and regulate dental papilla mesenchymal cell proliferation, differentiation, and dentinogenesis (Graf et al., 2016; Wu et al., 2016; Neves and Sharpe, 2018; da Silva Madaleno et al., 2020). YAP (Yes-associated protein) and transcriptional co-activator with PDZ-binding motif (TAZ) function as transcriptional cofactors of the Hippo pathway, which only indirectly bind to DNA and regulate target genes through interaction with other transcription factors such as Smads, Runx1/2, and their effectors of the transcriptional factor TEA domain (TEAD) family members (Piccolo et al., 2014). The cross talk between YAP/TAZ and BMP signaling has been documented. YAP was shown to support Smad1-dependent transcription and to be required for BMP-mediated suppression of neural differentiation in mouse embryonic stem cells, providing an example of long-term modulation of BMP signaling (Alarcón et al., 2009). Moreover, following stimulation with Bmp2, YAP modulated Smad1/5 phosphorylation and target gene expression in multiple cell types, while its early interaction with nuclear Smad1/5/8 was documented to lead to a stabilization of Smad1/5/8 signaling in astrocytes (Huang et al., 2016). Likewise, YAP overexpression activated Smad-dependent BMP signaling and upregulated the mRNA and protein expression of several cementogenesis markers including ALP, Runx2, osteocalcin (Ocn), and dentin matrix acidic phosphoprotein 1 (Dmp1). Treatment with a specific BMP antagonist (LDN193189) prevented the upregulation of the mRNA levels of ALP, Runx2, Dmp1, and Ocn as well as the intensity of ALP-stained and mineralized nodules in cementoblasts (Yang et al., 2018). The PPI study predicted that Bmp-induced proteins related to tooth/bone development interact with each other, and one protein binds to another *via* a third protein (Figure 5). It suggests that these proteins synergically regulate tooth-related gene expression mediated by Bmp2 signaling. Furthermore, the lncRNA–mRNA co-expression network analysis revealed that lncRNAs87211.1, lnc87189.1, and lnc86888.1 co-expressed with *Id2*, *Klf10*, *Lhx1*, *Smad7*, *Wnt1*, and *Wnt11* (Figure 6).

Based on the results of the KEGG, the PPI, and lncRNA–mRNA co-expression, the 9 mRNAs (*Fgf2*, *Id2*, *Id3*, *Lhx1*, *Smad7*, *Tcf7*, *Wnt1*, *Wnt11*, and *Klf10*) and 3 lncRNAs (87,211.1, 87,189.1, and 86,888.1) were selected as representative genes for RT-qPCR validation as these genes are involved in the dental cell proliferation and differentiation as well as tooth development and formation induced by Bmp2 signaling (Tsuboi et al., 2003; Koizumi et al., 2013; Sternberg et al., 2013; Chen et al., 2016; Graf et al., 2016; Wu et al., 2016; Li et al., 2019; Liu et al., 2019; Qin et al., 2019; Nottmeier et al., 2021) and three novel lncRNAs co-expressed with those mRNAs in the dental papilla cells. Our study demonstrated that the validated 9 mRNAs and 2 lncRNAs by RT-qPCR were consistent with the results of the RNA-seq, and the PCC method can be used to predict co-expression networks between mRNAs and lncRNAs.

The selected 9 mRNAs are involved in the dental cell proliferation, differentiation, and homeostasis (Ho et al., 2011; Koizumi et al., 2013; Sternberg et al., 2013; Bakopoulou et al., 2015; Chen et al., 2016; Zhang et al., 2016; Wang and Martin, 2017; Neves and Sharpe, 2018; Chen et al., 2019; Liu et al., 2019; Chakka et al., 2020; Niki et al., 2021). The loss of Bmp2 resulted in a decrease of these gene expressions detected by RNA-seq and RT-qPCR (Figures 2, 7). It suggested that Bmp2 regulates dental differentiation and dentinogenesis via these genes. It has been documented that the interplay of Bmp2 signal–associated mRNAs and lncRNAs regulates tooth development and formation (Liu et al., 2018; Zhong et al., 2020). This study showed that several novel lncRNAs such as lncRNA87211.1, lncRNA87189.1, and lncRNA86888.1 co-expressed with those mRNAs (Figure 6). However, whether the loss of Bmp2 downregulated expression of the selected 9 mRNAs through the lncRNA regulation needs to be further investigated although Liu’s and Zhong’s groups reported that lncRNAs inhibited microRNAs and indirectly upregulated Bmp2 gene expression, which enhanced the expression of Runx2, Dmp1, and Dspp genes as well as cell differentiation (Liu et al., 2018; Zhong et al., 2020).

Based on this study by the experimental and bioinformatics analyses, we suggest that Bmp2 signaling stimulates these mRNA expressions in the regulation of dental proliferation, differentiation, and homeostasis via direct or indirect lncRNA pathways. However, whether Bmp2 stimulates these 9 gene expressions *via* these lncRNAs during dentinogenesis needs to be further investigated.

Taken together, in the present study, we validated Bmp2 signal pathways and the possible signal molecules downstream of Bmp2 in the dental papilla cells using RNA-seq, RT-qPCR, and bioinformatics. Moreover, by performing bioinformatics analyses, we predicted several hub genes in the lncRNA–mRNA co-expression network regulated by Bmp2, which provides the direction and basis for further elucidating the regulation mechanism of Bmp2-mediated odontogenesis *via* the mRNA–lncRNA network.

## Data Availability

The authors acknowledge that the data presented in this study must be deposited and made publicly available in an acceptable repository, prior to publication. Frontiers cannot accept a article that does not adhere to our open data policies.
